# Development and Validation of a Questionnaire Assessing Fears and Beliefs of Patients with Knee Osteoarthritis: The Knee Osteoarthritis Fears and Beliefs Questionnaire (KOFBeQ)

**DOI:** 10.1371/journal.pone.0053886

**Published:** 2013-01-21

**Authors:** Mathilde Benhamou, Gabriel Baron, Marie Dalichampt, Isabelle Boutron, Sophie Alami, François Rannou, Philippe Ravaud, Serge Poiraudeau

**Affiliations:** 1 Service de Rééducation et Réadaptation de l'Appareil Locomoteur et des Pathologies du Rachis, Assistance Publique-Hôpitaux de Paris; Université Paris Descartes; INSERM IFR 25 Handicap, Paris, France; 2 Centre d'Epidémiologie Clinique, Assistance Publique-Hôpitaux de Paris; Université Paris Descartes, Paris, France; 3 Department of Sociology, Université Paris Descartes, Interlis, Paris, France; 4 Section Arthrose de la Société Française de Rhumatologie, Paris, France; University of South Australia, Australia

## Abstract

**Objective:**

We aimed to develop a questionnaire assessing fears and beliefs of patients with knee OA.

**Design:**

We sent a detailed document reporting on a qualitative analysis of interviews of patients with knee OA to experts, and a Delphi procedure was adopted for item generation. Then, 80 physicians recruited 566 patients with knee OA to test the provisional questionnaire. Items were reduced according to their metric properties and exploratory factor analysis. Reliability was tested by the Cronbach α coefficient. Construct validity was tested by divergent validity and confirmatory factor analysis. Test–retest reliability was assessed by the intra-class correlation coefficient (ICC) and the Bland and Altman technique.

**Results:**

137 items were extracted from analysis of the interview data. Three Delphi rounds were needed to obtain consensus on a 25-item provisional questionnaire. The item-reduction process resulted in an 11-item questionnaire. Selected items represented fears and beliefs about daily living activities (3 items), fears and beliefs about physicians (4 items), fears and beliefs about the disease (2 items), and fears and beliefs about sports and leisure activities (2 items). The Cronbach α coefficient of global score was 0.85. We observed expected divergent validity. Confirmation factor analyses confirmed higher intra-factor than inter-factor correlations. Test–retest reliability was good, with an ICC of 0.81, and Bland and Altman analysis did not reveal a systematic trend.

**Conclusions:**

We propose an 11-item questionnaire assessing patients' fears and beliefs concerning knee OA with good content and construct validity.

## Introduction

Fear is an emotional response generated during dangerous or painful experiences and can include potentially useful survival mechanisms such as escape and avoidance behaviours [1], [2]. After fearful experiences, anticipated or actual exposure to similar situations can re-elicit a fear response, even when these exposures are not dangerous or painful], a situation called classical conditioning. Fear can also be learned through vicarious exposure, including observing others (modelling) [3], and through information or instruction [4], [5]. Although emotion-based fear may be a relevant factor in some people, reason-based beliefs are important to all people. Beliefs are defined as convictions of the truth of propositions without their verification and therefore are subjective, mental interpretations derived from perceptions, reasoning or communications. All adults have measurable beliefs about diseases or their management that involve thoughts about the pathology or process responsible for the disease [6]. Beliefs are derived by processing information from multiple sources, including personal experiences, family, acquaintances, societal attitudes, media, literature, internet research, and encounters with the health care system [7]. Because human behaviours are shaped by beliefs, beliefs directly influence decisions to follow or not management strategies, including treatments. Of greatest importance, beliefs encompass ongoing reasoning and are therefore amenable to change in response to new information and new experiences [8]. Fears and beliefs have been mostly studied in patients with low back pain and have been shown to be important prognostic factors and to influence treatment adherence [9]–[13].

**Figure 1:**
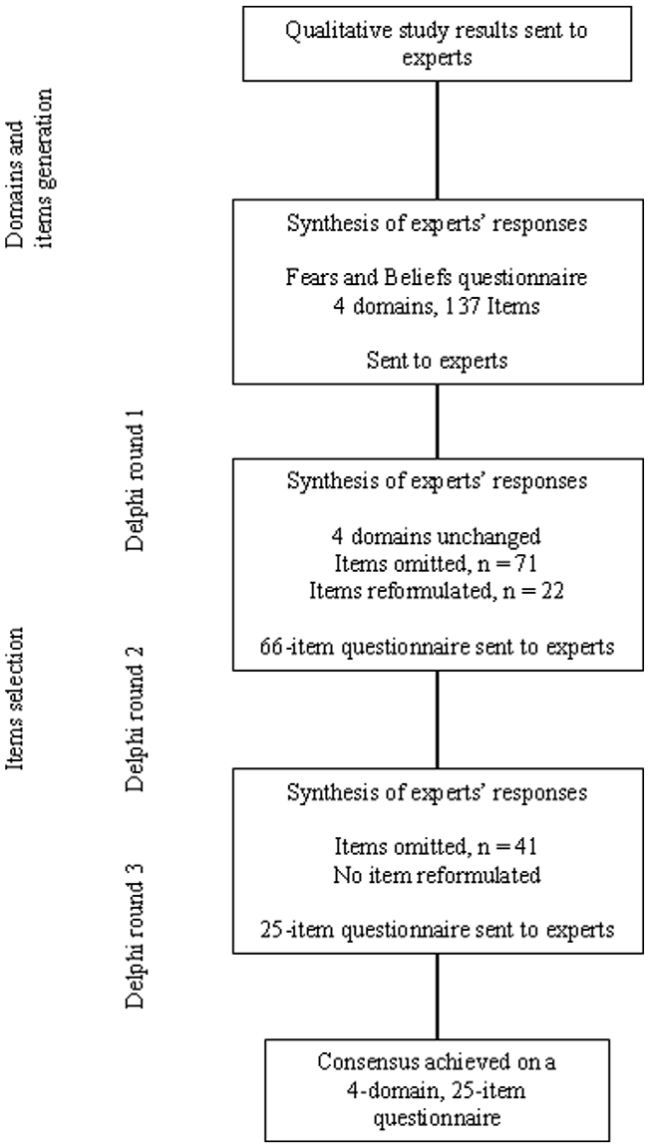
Development of the Knee Osteoarthritis Fears and Beliefs Questionnaire.

Arthritis (mainly osteoarthritis [OA]) is the most common cause of reported disability [14], [15] in developed societies and similar to chronic low back pain, is a chronic musculoskeletal painful situation. A recent survey suggested that the burden of knee OA in primary care is substantial [16], and a substantial decrease in health-related quality of life was also reported in a family practice setting [17]. Fears and beliefs of patients concerning knee OA management have been seldom studied. The Tampa scale of kinesiophobia (TSK) has been used in one cohort study and its score shown to be associated with psychological disability and walking at fast speed [18], and the metric properties of a brief fear of movement scale derived from the TSK have been recently published [19]. Although a modified version for knee OA of the fear-avoidance beliefs questionnaire has been used in one study [20], to our knowledge, no validated questionnaire has been specifically designed to assess fears and beliefs in OA patients.

We aimed to develop a questionnaire assessing fears and beliefs of patients with knee OA: the Knee Osteoarthritis Fears and Beliefs Questionnaire (KOFBeQ).

## Methods

The general methodology used to develop this questionnaire have been previously published [21].

### 1) Development of the provisional questionnaire

We adopted a Delphi procedure to select items for the provisional questionnaire. We used previously described general methods for instrument development [22]. The process consists of 3 main steps: definition of the aim of the questionnaire, generating items, and selecting items.

### Aim of the questionnaire

The general purpose of this questionnaire is to facilitate the patient–physician relationship and patient education by recording patient fears and beliefs in routine practice and clinical research. The specific purposes are to better define patients' unrealistic fears and beliefs to try to modulate barriers to treatment adherence and help plan disease management.

### Generating items

The Delphi consensus method was used to generate and select items [22], with the initial development in French. For extracting items related to fears and beliefs, a detailed document reporting on the qualitative analysis of interviews with patients was sent to 10 experts (1 general practitioner, 5 rheumatologists, 1 sociologist, 1 orthopaedic surgeon, 1 physical therapist, and 1 physical and rehabilitation medicine physician) [23]. Experts were asked to read the documents and extract the most relevant items concerning patient fears and beliefs. To help experts, several domains were proposed: the disease, its causes and outcomes (triggering and worsening factors); impact of knee OA on daily living, sports, leisure and professional activities; treatments; and physicians. Experts were invited to add domains if they wished.

### Selecting items

For each generated item, experts were asked to rate on two 11-point Likert scales (0, disagree, to 10, agree) whether they believed the item should be selected in the final tool and the degree of agreement with the formulation of the item. Experts who disagreed with the formulation of the item were asked to propose a new formulation. Experts were also invited to add items to domains. Items with median relevance score ≤7 were excluded, as were redundant items.

For the second Delphi round, experts were asked to re-rank their agreement with each item; they could change their score in view of the group's response to the previous round but could not conform to the group's median response. A rewording of some items was proposed. Further, an explanation for the questionnaire and the modalities of answers were developed. Items with median relevance score ≤8 were excluded. A rewording of some items was proposed.

During the third and last round, experts commented on the final checklist and modalities of answers. Agreement was obtained with the third Delphi round.

### Analysis of items

The responses for each Delphi round were reported as the percentage of experts choosing each value of the 11-point Likert scale. Experts' comments on each item were recorded. After each round, the steering group (IB, SA, SP) discussed experts' qualitative and quantitative answers. From these answers, redundant items were combined, categories of items with insufficient consensus rates were excluded, items proposed by experts were added, and items were modified or expanded.

### English translation of the provisional questionnaire

To provide a version of the questionnaire for English-speaking patients, the French version was translated by the forward and backward translation procedure [24], [25]. Two independent bilingual translators, whose native language was English, translated the French version of the questionnaire into English. As recommended, the translators were encouraged to strive for idiomatic rather than word-for-word translation. Two bilingual investigators (SP, FR) compared the 2 translated versions, with consensus. Two other independent translators who had not participated in the first stage and whose native language was French then back-translated the English version of the questionnaire into French. The investigators (SP, FR) then compared the translated version, with consensus.

### 2) Reduction of items in the provisional questionnaire and validation of the final questionnaire

The aim was to select items with the best metric qualities from the provisional questionnaire and to assess the reliability and construct validity of the final questionnaire. Therefore, we conducted a national multicenter cross-sectional survey of patients in a primary care setting.

### Recruitment of physicians and patients

#### Physicians

Rheumatologists and general practitioners (GPs) were randomly selected from 2 national databases of 475 and 68 594 practitioners, respectively, who had not previously refused to participate in studies or surveys. The assigned physicians were contacted by mail, then telephone calls if they did not respond. The randomisation was stratified by geographic area. Each physician was asked to include 5 consecutive patients.

#### Patients

Each patient consulting one of the participating physicians for knee OA during the period of inclusion and meeting the inclusion criteria was asked to participate in the study.

The inclusion criteria were age 45 years and older, knee OA defined by the American College of Rheumatology criteria [26], whatever knee OA activity status or treatment used, and written consent to participate in the study. The exclusion criteria were absence of knee radiographs and inability to complete a questionnaire.

Patients were included from September 2009 to March 2010.

### Data collection

Data recorded were patient characteristics: socio-demographic (age, sex, marital status, level of education, employment status, living area), medical (body mass index, duration of disease, co-morbidities, type of knee OA [medial femoro-tibial, lateral femoro-tibial, femoro-patellar]), level of physical activity, pharmacological and physical treatments for OA, and OA activity and function (pain intensity on an 11-point numeric scale [0–10], physician opinion of severity on an 11-point numeric scale [0–10], WOMAC functional scale, and SF-12 physical and mental scales). The questionnaires were completed on paper or electronically according to patient preference.

### Sample size calculation

We expected a Cronbach α coefficient of 0.7 to 0.9 for the KOFBeQ. We needed to include 400 patients for a coefficient of 0.7 with 0.05 accuracy and one of 0.9 with 0.015 accuracy. This number of 400 patients was also sufficient for excellent accuracy of the coefficients on factorial analysis.

We assumed that each physician would include 5 patients. Therefore, we planned to enrol 80 physicians (80% [64] GPs, and 20% [16] rheumatologists). We hypothesized that 25% of the physicians contacted would agree to participate and that 75% of these would include patients.

### Statistical analysis

We used descriptive statistics to examine the response distribution to each item. The scoring system was 10-points Likert scale (0–9). Items with the following characteristics were removed: low response rate (≤95%); floor or ceiling effect, defined by more than 50% of the respondents choosing an extreme positive or negative response category, respectively; and high inter-item correlation (>0.70) assessed by Spearman correlation coefficient.

We used explanatory factor analyses with principal component analysis (PCA) to examine the construct validity of the KOFBeQ. Oblique promax rotation was selected because the factors were not expected to be completely independent of each other [27]. Factors generated by PCA were extracted if eigenvalues were greater than the randomly generated factors from Horn's parallel analysis [28]. Items were included in the factors if they revealed loadings greater than 0.5. In the case of multiple loading of an item on several factors, the item was included in the factor with a better conceptual relationship.

We assessed the internal consistency of the KOFBeQ by the Cronbach α coefficient to examine the degree to which the items in a scale measured the same concept [29]; a Cronbach α >0.70 was considered acceptable, 0.71 to 0.80 respectable, and >0.8 very good. The 95% confidence interval (95% CI) of the Cronbach α was assessed by the bootstrap technique with 1000 replications.

For confirmatory factor analysis, the multi-trait method was used to test the significantly higher correlation of each item with items of its hypothesized factor than with items of the other factors [27] in a different sample of 40 patients. Distributions of intra- and inter-factor correlations were compared by a boxplot graphic.

Divergent validity was assessed by Spearman correlation of the global score of the KOFBeQ and other outcome measures (knee OA severity assessed by physicians, knee pain, function WOMAC score and SF-12 physical and mental scores).

For test-retest reliability, patients from the cross-sectional survey could not participate because a visit to the physician might modify expectations. Therefore, we selected a sample of 40 patients from the files of the physical and rehabilitation medicine department and mailed them a questionnaire to complete at 2-week intervals. Test–retest reliability was assessed by the intra-class correlation coefficient (ICC) by a two-way random-effects model (an ICC ≥0.75 is considered excellent reproducibility) [30] and the Bland and Altman method, by calculating the mean difference (δ) between 2 measurements and the standard deviation (SD) of the difference [31]. The 95% limits of agreement were defined as the mean difference between the measurements ±1.96 SD of the differences. By definition, if differences are normally distributed, 95% of individual differences are within 2 SD of the mean difference (i.e., within the limits of agreement). The Bland-Altman plot is useful to search for any systematic bias, assess random error and reveal whether the difference between scores depends on the level of scores. We computed the standard error of measurement (SEM: essentially, the average SD among observations from the same subject) [31]. The SEM was estimated by calculating the square root of the within-subject variance (SEM = √σ_between measurement_ + σ_residual_). From the same set of data, the smallest detectable change (SDC) was calculated by the formula SDC  = 1.96 * √2 * SEM. SDC allows to be 95% confident that the observed change is a real change.

Data analysis involved use of R 2.10.1 and SAS 9.1.

### Ethical considerations

All patients gave their written informed consent to participate in the study. The study protocol was approved by the ethics committee of Cochin Hospital, Paris. Investigations were conducted according to the principles of the Declaration of Helsinki.

## Results

### 1) Development of the questionnaire assessing fears and beliefs of patients with knee OA (Figure 1)

The experts did not generate new domains for the questionnaire. Synthesis of experts' responses to the analysis of the qualitative study led to the extraction of 137 items concerning fears and beliefs.

#### Selection of items

For the first Delphi round, the 137-item questionnaire was mailed to experts. Experts' responses were synthesized, items with median relevance ≤7 were eliminated, and redundant items were combined, for a 66-item questionnaire. We reformulated 22 items. Domains were combined into 4 categories: causes and evolution of the disease, impact on daily activities, treatments, and physicians.

For the second Delphi round, the 66-item questionnaire was sent back to experts, along with the median scores for relevance and quality of the formulation of each item obtained during the first round, with minimum and maximum scores. Experts were asked again to rate the relevance and quality of the formulation of each item on two 11-point scales. After synthesis of experts' responses, a 25-item questionnaire assessing patient fears and beliefs for knee OA management (Appendix S1) was sent to experts for the third Delphi round. Consensus was achieved at this stage.

We identified few discrepancies between each translation by the forward and backward translation procedure, and consensus was easily reached. Therefore, the translated versions and the original versions explored the same dimensions.

### 2) Reduction of items and validation of the questionnaire

#### Patients

**Figure 2:**
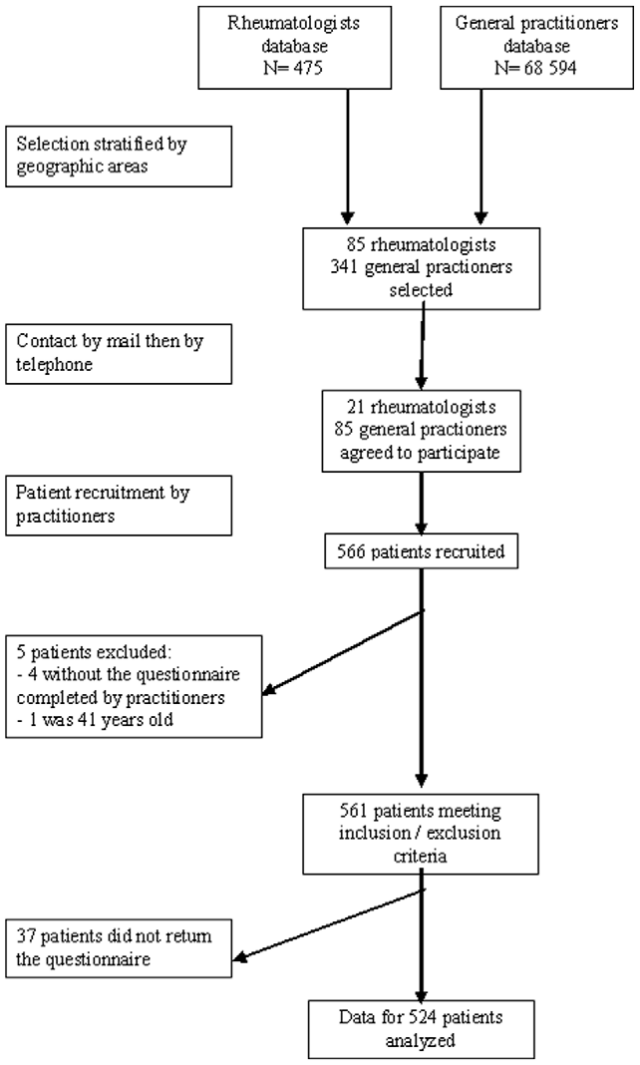
Flow chart of practitioners through the trial.

Physicians recruited 566 patients to test the questionnaire (Figure 2). Five patients were excluded (4 because the physician questionnaire could not be retrieved for verification of inclusion and exclusion criteria and 1 because he was 41 years old), 37 patients did not return their questionnaire; finally, data for 524 patients were analysed.

The mean (SD) age of patients was 68.2 (10.1) years, disease duration 6.6 (5.3) years, pain intensity 5.6 (2.1; 0–10 scale), and WOMAC score 31.8 (12.9, range 1–62) (Table 1).

**Table 1:** Characteristics of patients with knee osteoarthritis (OA) surveyed in developing the Knee Osteoarthritis Fears and Beliefs Questionnaire.

Sociodemographic characteristics	N	
Age, years, mean (SD)	524	68.2 (10.1)
Female sex	524	327 (62.5)
Married	524	294 (56.4)
Level of education	524	
- primary		272 (52.4)
- secondary		171 (32.9)
- post-graduate		76 (14.6)
Employment status	524	
- job activity		96 (18.4)
- retired		374 (71.8)
- no job activity		29 (5.6)
- unemployed		4 (0.8)
- invalidity		18 (3.5)
Living area	524	
- rural		210 (41.4)
- urban		297 (58.6)
Level of physical activity	524	
- professional sports activity		12 (2.3)
- intensive sports activity		13 (2.5)
- regular sport activity		69 (13.2)
- occasional sport activity		88 (16.8)
- no sport activity		342 (65.3)
**Medical characteristics**		
Body mass index, kg/m^2^, mean (SD)	520	28.3 (4.9)
Duration of disease, years, mean (SD)	520	6.6 (5.3)
Co-morbidities	524	
- cardiovascular abnormality		293 (55.9)
- metabolic and endocrinal disorders		166 (31.7)
- joint and bone disorders (except knee OA)		48 (9.2)
- gastrointestinal disorders		72 (13.7)
- respiratory function		35 (6.7)
Medial femoro-tibial knee OA	524	219 (52.9)
Lateral femoro-tibial knee OA	524	73 (39.5)
Femoro-patellar knee OA	524	145 (53.1)
Physician scale of severity of knee OA (0–10), mean (SD)	523	5.9 (1.8)
Pain intensity (0–10), mean (SD)	405	5.6 (2.1)
Medical drugs for OA		
- analgesics	496	459 (92.5)
- nonsteroidal anti-inflammatory drugs	475	234 (49.2)
- slow-acting drugs for OA	484	312 (64.5)
Physical treatments for OA		
- exercise	455	126 (27.7)
- physiotherapy	466	153 (32.8)
- alternative medicine	458	46 (10.0)
**Functional status**		
WOMAC score, mean (SD), range	476	31.8 (12.9), 1–62
SF-12, mean (SD)	461	
-physical score (range 0–100)		35.4 (8.0)
-mental score (range 0–100)		44.4 (10.3)

Data are number (%) unless indicated.

The 38 patients not included in the validation of the questionnaire were somewhat younger (5 years younger, on average), had less disease duration (28.9% vs. 45.5% had OA for more than 5 years), and were taking more nonsteroidal anti-inflammatory drugs (66.7% vs. 49.2%) than patients whose data were analyzed (data not shown).

### Item reduction

In total, data for 524 patients were analyzed at this step. Concerning the 25-item provisional questionnaire, no missing values occurred for 455 cases (86.8%).

Fourteen items were omitted after the item reduction process, for an 11-item questionnaire (score range 0–99, Appendix S2). We omitted 1 item with a non-response rate >5% (item 16 of the provisional questionnaire, Appendix S1). We omitted 1 item with a ceiling effect (item 3 of the provisional questionnaire, Appendix S1); no item had a floor effect. Two pairs of items were highly correlated (Spearman correlation coefficients 0.7–0.8) and 2 items were omitted (items 4 and 11 of the provisional questionnaire, Appendix S1).

**Figure 3:**
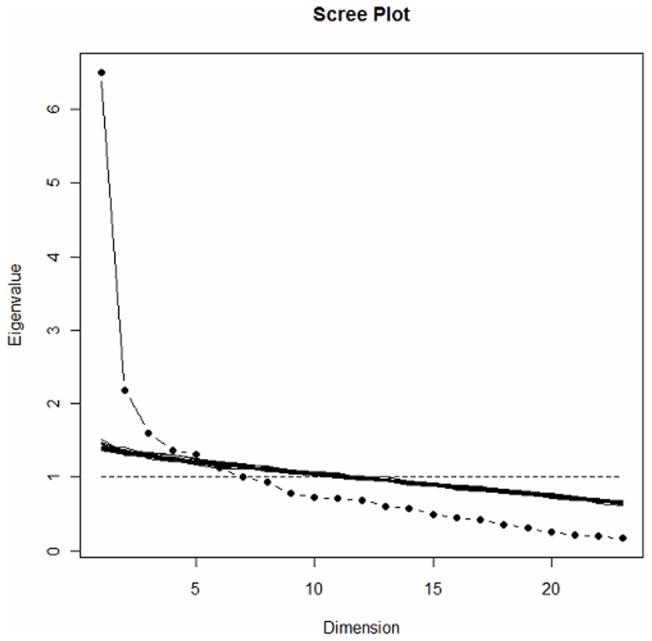
Exploratory factor analysis of the Knee Osteoarthritis Fears and Beliefs Questionnaire.

Exploratory factor analysis extracted 4 main factors with eigenvalues of 6.51, 2.19, 1.60, and 1.37 explaining 41% of the variance (Figure 3). Each factor was easily characterized, factor 1 (3 items) representing fears and beliefs about daily living activities, factor 2 (4 items) fears and beliefs about physicians, factor 3 (2 items) fears and beliefs about the disease, and factor 4 (2 items) fears and beliefs about sports and leisure activities. Another 10 items (items 1, 2, 7–9, 17–20, and 22 of the provisional questionnaire, Appendix S1) were eliminated because of weak correlation (<0.5) for each factor.

### Validity of the questionnaire

**Figure 4:**
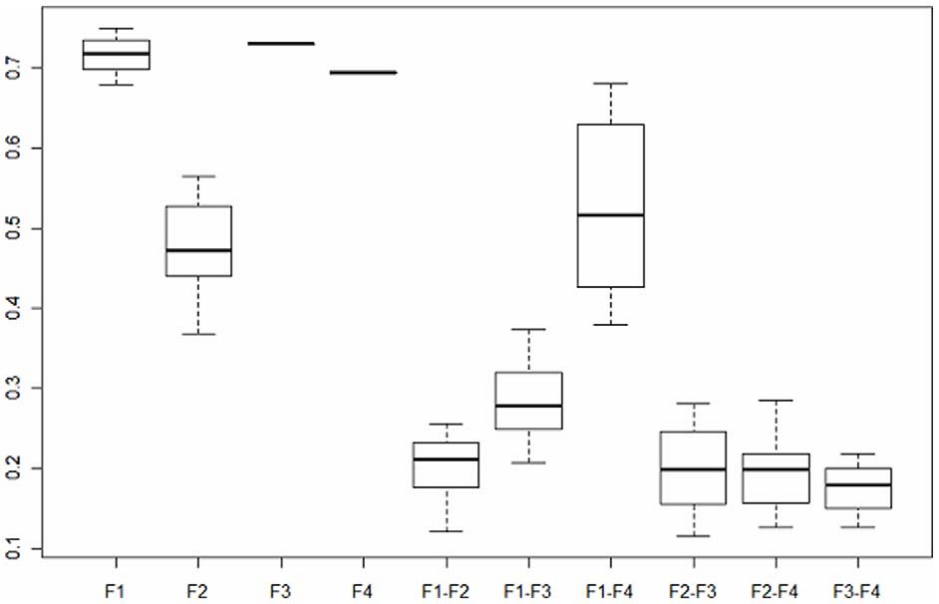
Confirmatory factor analysis of the Knee Osteoarthritis Fears and Beliefs Questionnaire.

Overall reliability was excellent, with a Cronbach α coefficient of 0.85 (95% CI 0.83–0.87). Reliability of each factor was good, with Cronbach α coefficients of 0.89 (0.80–0.89) for factor 1, 0.78 (0.74–0.82) for factor 2, 0.85 (0.80–0.89) for factor 3, and 0.84 (0.80–0.87) for factor 4. Confirmatory multi-trait analyses confirmed higher intra- than inter-factor correlations (Figure 4).

Expected divergent validity was observed with knee pain score (r = 0.38), WOMAC function score (r = 0.52), and physical and mental component scores of the SF-12 (r = 0.36 and r = 0.38, respectively) (table 2).

**Table 2:** Divergent validity: correlation of the global score of the Knee Osteoarthritis Fears and Beliefs Questionnaire with other outcome measures.

	Spearman correlation coefficients
Knee OA severity (0–10) assessed by physicians	0.30
Knee pain (0–10)	0.38
Function WOMAC score	0.52
SF-12 physical score	−0.36
SF-12 mental score	−0.38

### Test-retest validity

**Figure 5:**
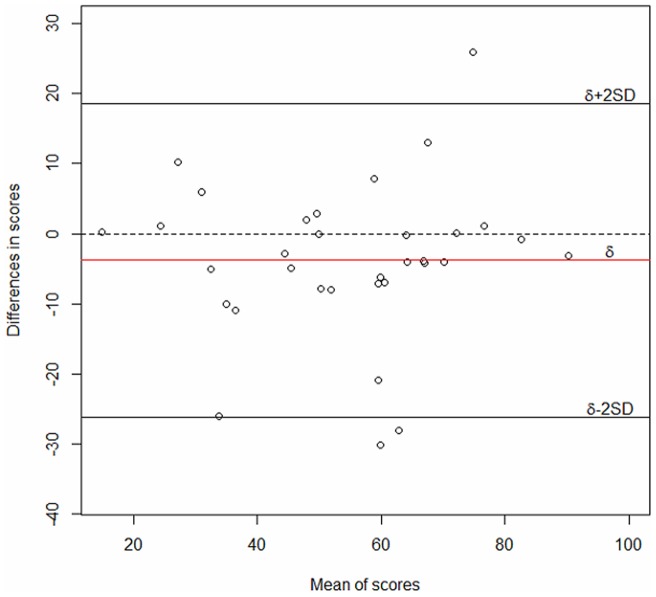
Bland and Altman analysis of test–retest reliability.

Test–retest reliability was good (table 3), with an ICC of 0.81 (95% CI 0.64–0.90), and Bland and Altman analysis revealed a slight bias in mean differences (−3.79 [1.95]) without a systematic trend (Figure 5). The variability was random and uniform throughout the range of values (Figure 5). With the limits of agreement, 95% of the differences between the 2 measurements could be expected to lie between −26.2 and 18.6 points (2 SD of the mean difference). From the value of the SEM, the SDC for the global score was 22.8 points, which in a maximum of 99 points equates to a 23.0% score change.

**Table 3:** Test-retest reliability for the global score.

	N	Mean baseline (SD)	Mean 2 weeks (SD)	Δ mean (SD)	Limits of agreement	ICC (95%CI)	SEM	SDC
Global score	33	52.4 (19.0)	56.2 (18.2)	−3.79 (1.95)	−26.2 to 18.6	0.81 (0.64–0.90)	8.2	22.8

SD  =  Standard Deviation, ICC  =  intraclass correlation coefficient for agreement, CI  =  Confidence Interval, SEM  =  Standard Error of Measurement, SDC  =  Smallest Detectable Change.

### Scoring of the KOFBeQ

The principal component analysis showed a first eigenvalue substantially higher than the others explaining 28.3% of the variance. According to the latter result and those of reliability analyses, an overall score can be used (0–99). The global score is obtained by adding scores of each of the 11 items. The metric properties of the global score should be tested in another sample of patients with knee OA.

## Discussion

To our knowledge, this is the first questionnaire assessing patients' fears and beliefs concerning knee OA. For musculoskeletal conditions, fears and beliefs have been mainly studied in low back pain and shown to be important predictors of severity and outcome [13]. Therefore, measuring fears and beliefs in patients with knee OA may help in developing treatment approaches such as education and behavioral therapy and better define prognosis at the individual level.

The main strength of this study is probably the design adopted to generate items. The in-depth interviews about views of patients with knee OA and its management provide a relevant qualitative database to select items that really matter to the patient when building a patient-reported questionnaire. This approach is strongly recommended by the US Food and Drug Administration [32] because it increases the content validity of the instrument. Most patient-reported outcomes widely used with OA were developed in the 1980s and 1990s mainly by selecting items from expert viewpoints and/or pre-existing questionnaires [33]–[37]. Because patient and physician views differ on what is important or what matters [38]–[40], the content validity of these questionnaires is questionable. Another strength is that the sample is likely to be representative of patients with knee OA in primary care. Physicians were asked to include 5 consecutive patients and demographic and clinical characteristics of the patients are similar of those previously published in a primary care French context [16].

The Delphi design adopted to select items from the qualitative study is a classical recommended method [22] that allows experts to give an opinions blinded to the opinions of other experts in a first step and achieve consensus anonymously in a second step. The method prevents a “leading expert” effect. Items generated in the provisional questionnaire we developed seemed understandable and acceptable in view of the very low rate of missing answers to the provisional scale.

We translated the provisional questionnaire in English rather than the final one to let researchers from other English-speaking countries test the item reduction step in their own country if they feel it could be relevant in a different background.

The Delphi procedure preserved the 4-domain structure that the steering group proposed to help experts select items: the disease, its causes and outcomes (triggering and worsening factors); impact of knee OA on daily living, sports, leisure and professional activities; treatments; and physicians. Therefore the final questionnaire explores important domains of fears and beliefs that may have an impact on the burden of the disease and its management.

The metric performances of the questionnaire are promising. It has excellent internal validity (reliability) and test–retest reproducibility. It is likely to have satisfactory construct validity because we observed the expected divergent validity, and the factorial structure seems robust, with 4 factors identified and easily characterized after exploratory factor analysis and confirmed by confirmatory factor analysis. Patient fears and beliefs are organized around 4 axes: daily living activities; physicians; the disease; and sports and leisure activities.

This study has limitations. The main limitation is that we did not include patients in the group of experts for the Delphi procedure. Although knee OA is a frequent clinical situation, a patient association is lacking in France, and the identification and selection of patients implicated in the disease and its management is far from obvious. Furthermore, we did not test face validity of the final 11-item questionnaire with a different sample of patients. Another limitation is that this questionnaire has been developed in a strict French context and its content validity should be verified in other groups of patients with different cultural backgrounds. However, the French society is a highly multicultural one, and this limitation applies to every patient-reported outcome because none of them has been developed simultaneously in different countries with different languages and cultures. For assessment of the validity of the questionnaire, we assessed divergent validity but not convergent validity. However, no other instrument exists to assess fears and beliefs or a concept close to fears and beliefs in this context.

Finally, use of this questionnaire may be helpful for two different approaches: a qualitative individualized analysis of responses in routine practice may help increase the quality of patient education by providing relevant information to physicians to adapt attitudes, educational messages, and treatment strategies according to patient fears and beliefs, and a quantitative analysis may provide useful information in clinical research into the effect of high or low level of fears and beliefs or their modification on compliance with treatment, outcomes of treatments, and disease evolution. In this perspective, sensitivity to change of the instrument should be first demonstrated.

In conclusion, we propose a new 11-item patient self-reported questionnaire assessing patient fears and beliefs about knee OA. This questionnaire has robust metric properties, particularly content and construct validity. Its usefulness in the clinic and in clinical research remains to be demonstrated.

## Supporting Information

Appendix S1 The provisional questionnaire assessing fears and beliefs of patients with knee osteoarthritis for disease management.
(DOC)

Appendix S2 The Knee Osteoarthritis Fears and Beliefs Questionnaire (KOFBeQ).
(DOC)
