# Supplementary material for: Development and Validation of a Questionnaire Assessing Fears and Beliefs of Patients with Knee Osteoarthritis: The Knee Osteoarthritis Fears and Beliefs Questionnaire (KOFBeQ)
Source: PLoS One. 2013 Jan 21;8(1):e53886. doi: 10.1371/journal.pone.0053886 (PMC3549996; doi:10.1371/journal.pone.0053886)
Supplement: Appendix S1 — The provisional questionnaire assessing fears and beliefs of patients with knee osteoarthritis for disease management. (DOC) [file pone.0053886.s001.doc]

**APPENDIX**

Appendix 1: The provisional questionnaire assessing fears and beliefs of patients with knee osteoarthritis for disease management

### These are statements that other patients have expressed about their knee osteoarthritis. For each statement, evaluate to what extent you agree or not with the statement.

1- Old age is the main cause of knee osteoarthritis

2- Knee osteoarthritis is caused by cartilage wear

3- Traumas are the main cause of knee osteoarthritis

4- Knee osteoarthritis cannot be cured

5- Nothing can be done to modify the evolution of knee osteoarthritis

6- Knee osteoarthritis is a fate for which not much can be done

7- Physical activities are harmful for knee osteoarthritis

8- Wet weather makes knee osteoarthritis worse

9- Some diets or food supplementations can prevent knee osteoarthritis

10- Because of my knee OA, I will soon be unable to walk any more

11- Because of my knee OA, I will end up in a wheelchair

12- Because of my knee OA, I will have to give up my leisure activities

13- Because of my knee OA, I will have to stop sport activities

14- Because of my knee OA, I will depend on others for activities of daily living

15- Because of my knee OA, I will not be able to climb stairs anymore

16- Knee infiltrations can damage knee cartilage

17- Using a cane gives the image of very old age

18- Exercises are useless for knee osteoarthritis

# 19- Medications are not helpful for knee osteoarthritis

# 20- Knee surgery is inevitable when you have knee osteoarthritis

21- Physicians are not interested in knee osteoarthritis

22- Having a physician who prescribes exams for knee osteoarthritis makes you more secure

23- General practitioners usually do not refer patients with knee osteoarthritis to specialists because knee osteoarthritis is a common and benign disease

24- Physicians underestimate pain in knee osteoarthritis

# 25- Physicians do not have much to propose for knee osteoarthritis
