# Supplementary material for: Development and Validation of a Questionnaire Assessing Fears and Beliefs of Patients with Knee Osteoarthritis: The Knee Osteoarthritis Fears and Beliefs Questionnaire (KOFBeQ)
Source: PLoS One. 2013 Jan 21;8(1):e53886. doi: 10.1371/journal.pone.0053886 (PMC3549996; doi:10.1371/journal.pone.0053886)
Supplement: Appendix S2 — The Knee Osteoarthritis Fears and Beliefs Questionnaire (KOFBeQ). (DOC) [file pone.0053886.s002.doc]

**Appendix S2 : The Knee Osteoarthritis Fears and Beliefs Questionnaire (KOFBeQ)**

### These are statements that other patients have expressed about their knee osteoarthritis. For each statement, evaluate to what extent you agree or not with the statement.

#### **Daily living activities**

1- Because of my knee OA, I will soon be unable to walk

2- Because of my knee OA, I will depend on others for activities of daily living

3- Because of my knee OA, I will not be able to climb stairs anymore

#### **Physicians**

4- Physicians underestimate pain in knee osteoarthritis

# 5- Physicians do not have much to offer for knee osteoarthritis

6- General practitioners usually do not refer patients with knee osteoarthritis to specialists because knee osteoarthritis is a common and minor disease

7- Physicians are not interested in knee osteoarthritis

**Disease**

8- Knee osteoarthritis is a fate for which not much can be done

9- Nothing can be done to modify the progression of knee osteoarthritis

**Sports**

10- Because of my knee OA, I will have to stop sport activities

11- Because of my knee OA, I will have to give up my leisure activities

Response modalities

| 0 1 2 3 4 5 6 7 8 9  Totally agree Totally disagree |
| --- |
